# The use of a biphasic calcium phosphate in a maxillary sinus floor elevation procedure: a clinical, radiological, histological, and histomorphometric evaluation with 9- and 12-month healing times

**DOI:** 10.1186/s40729-017-0099-x

**Published:** 2017-07-25

**Authors:** W. F. Bouwman, N. Bravenboer, J. W. F. H. Frenken, C. M. ten Bruggenkate, E. A. J. M. Schulten

**Affiliations:** 1 Department of Oral and Maxillofacial Surgery/Oral Pathology, VU University Medical Center/Academic Centre for Dentistry Amsterdam (ACTA), P.O. Box 7057, 1007 MB Amsterdam, The Netherlands; 2 Department of Clinical Chemistry, VU University Medical Center, Amsterdam, The Netherlands; 3 Department of Oral and Maxillofacial Surgery, St. Antonius Hospital, Nieuwegein, The Netherlands; 4 Department of Oral and Maxillofacial Surgery, Alrijne Hospital, Leiderdorp, The Netherlands; 5 Department of Oral and Maxillofacial Surgery, The Tergooi Hospital, Blaricum, The Netherlands

**Keywords:** Biphasic calcium phosphate, Bone substitute, Sinus augmentation, Sinus floor elevation

## Abstract

**Background:**

This study evaluates the clinical, radiological, histological, and histomorphometric aspects of a fully synthetic biphasic calcium phosphate (BCP) (60% hydroxyapatite and 40% ß-tricalcium phosphate), used in a human maxillary sinus floor elevation (MSFE) procedure with 9- and 12-month healing time.

**Methods:**

A unilateral MSFE procedure, using 100% BCP, was performed in two series of five patients with healing times of 9 and 12 months respectively. Clinical and radiological parameters were measured up to 5 years postoperatively. Biopsy retrieval was carried out during dental implants placement. Histology and histomorphometry were performed on 5-μm sections of undecalcified bone biopsies.

**Results:**

The MSFE procedure with BCP showed uneventful healing in all cases. All dental implants appeared to be well osseointegrated after 3 months. Radiological evaluation showed less than 1 mm tissue height loss from MSFE to the 5-year follow-up examination. No signs of inflammation were detected on histological examination. Newly formed mineralized tissue was found cranially from the native bone. The BCP particles were surrounded by connective tissue, osteoid islands, and newly formed bone. Mineralized bone tissue was in intimate contact with the BCP particles. After 12 months, remnants of BCP were still present. The newly formed bone had a trabecular structure. Bone maturation was demonstrated by the presence of lamellar bone. Histomorphometric analysis showed at 9 and 12 months respectively an average vital bone volume/total volume of 35.2 and 28.2%, bone surface/total volume of 4.2 mm^2^/mm^3^ and 8.3 mm^2^/mm^3^, trabecular thickness of 224.7 and 66.7 μm, osteoid volume/bone volume of 8.8 and 3.4%, osteoid surface/bone surface (OS/BS) of 42.4 and 8.2%, and osteoid thickness of 93.9 and 13.6 μm.

**Conclusions:**

MFSE with BCP resulted in new bone formation within the augmented sinus floor and allowed the osseointegration of dental implants in both groups. From a histological and histomorphometric perspective, a 9-month healing time for this type of BCP may be the optimal time for placement of dental implants.

## Background

Maxillary sinus floor elevation (MSFE) is a surgical procedure to enhance the bone height in the posterior maxilla with graft material, allowing dental implant placement (later or at the same time) [1, 2]. This pre-implant procedure is predictable and results in a dental implant survival of more than 93.8% 3 years after dental implant placement [3]. According to Pjetursson [4] in his systematic review on success of implants inserted in combination with sinus floor elevation, the implant survival increases to 98.3% after 3 years when compared to non-augmented jawbone.

Autogenous bone is still the gold standard, because of its osteoconductive and osteoinductive properties, due to the possible osteogenic capacity [5–10]. Moreover, the bone morphogenic proteins, present in autogenous bone grafts, can attract osteogenic cells from the surrounding tissues, in their turn containing other growth factors essential for the process of bone graft incorporation [4].

As the maxillary tuberosity, mandibular retromolar or chin region do not always supply enough bone graft volume, bone grafts can also be harvested from the anterior iliac crest, the tibia, the rib, and the calvarian bone. However, these harvesting procedures have disadvantages, such as prolonged operating time, donor site morbidity, hospitalization [9, 11–13], sensory disturbances [14], and unpredictable resorption rate of the bone grafts [5, 15]. Donor site morbidity may be a major reason to question the use of autogenous bone [16]. Therefore, several types and properties of bone substitutes (alloplast, xenograft, allograft, and mixtures of various materials) have been developed [16, 17] to overcome the disadvantages mentioned above.

Calcium phosphates, such as hydroxyapatite (HA), β-tricalcium phosphate (β-TCP), or biphasic calcium phosphate (BCP), a mixture of HA and β-TCP, are osteoconductive as they resemble the chemical composition of natural bone [18, 19]. Calcium phosphates are biocompatible and do not induce a sustained foreign body response or toxic reaction [20]. At a physiological pH, calcium phosphates are the least soluble of the naturally occurring calcium phosphates, which makes them relatively resistant to resorption [21–23].

β-TCP is a biocompatible osteoconductive calcium phosphate that may provide a scaffold for potential bony ingrowth [24]. β-TCP resorbs rather quickly but not necessarily at the same rate as new bone formation [25–27]. Most research focused on either using the relative unresorbable HA as a scaffold or β-TCP as a degradable component [19, 24–26, 28, 29]. Zerbo et al. [30] concluded that due to the absence of osteoinductive properties of TCP, the rate of bone formation was delayed in comparison with autogenous bone grafts. It would be beneficial for the patient to reduce the interval between the MSFE procedure and dental implant placement to accelerate the process of integration of the grafted material. BCP, in a combination of 60% HA and 40% β-tricalcium phosphate, demonstrated new bone formation in both animals and humans [24, 31–33]. This biphasic calcium phosphate (BCP) appeared to be a suitable graft material for vertical augmentation of the posterior maxilla by means of an MSFE procedure and dental implants placement in a study with a healing time of 6 months [16, 27, 34].The process of bone substitution may not be completed after 6 months of follow-up [27, 35]. Even though clinically, the tissue seems stable enough for dental implant placement, the high bone formation, especially in the newly formed bone areas, indicates that after 6 months, bone cells are still actively replacing BCP in vital bone tissue. To date, no long-term follow-up has been reported on the use of a synthetic BCP, consisting of 60% HA and 40% β-TCP, which may elucidate the degradation properties of BCP material. One may have to consider that more time is necessary to achieve a new bone balance. The aim of this study is to evaluate the clinical, radiological, histological, and histomorphometric aspects of a synthetic BCP (Straumann® Bone Ceramic, Institut Straumann AG, Basel Switzerland) that was used in a MSFE procedure with 9- and 12-month healing times.

## Methods

### Study population

In this study, 10 consecutive healthy patients were selected for a unilateral MSFE procedure. Five patients received dental implants 9 months after MSFE and five patients underwent dental implant surgery 12 months after MSFE. In the 9-month group (three men and two women), the average age was 56.6 years (range 40 to 64 years); in the 12-month group (one man and four women), the average age was 58.2 years (range 51 to 67 years). All patients were partially edentulous in the posterior maxilla without the need for onlay bone grafting of the alveolar crest to achieve an adequate alveolar ridge. A minimal native bone height of 4 mm (calculated from measurements on a preoperative panoramic radiograph) was preferred in both study groups. All selected patients were non-smokers, showed no systemic disease, and were not drug users.

The study was performed in accordance with the principles of the Declaration of Helsinki. Since the study involved CE-marked devices (calcium phosphates) being used for their intended purpose (use as carrier material for bone augmentation in sinus floor elevation procedures) and the harvested material can be regarded as surgical waste, no specific regulatory approval from a medical ethical committee was required. Patients provided written consent before the study-related procedures were undertaken. The biopsies were retrieved during dental implant surgery by means of trephine drills, implicating the tissue in the hollow drill is considered surgical waste. For the patient, this is not an additional invasive procedure. The different healing times did not have a negative impact on the patients.

### Maxillary sinus floor elevation procedure

Ten patients were scheduled for a unilateral two-stage MSFE top-hinge door lateral window technique procedure, as described by Tatum [2]. All 10 patients were treated in an outpatient procedure under local anesthesia. Perioperatively, all patients received an antibiotic profylaxis, consisting of amoxicillin 500 mg four times daily for 7 days, starting 1 day before the MSFE procedure. An oral rinse with chloorhexidine-digluconate 0.12%, three times, 10 cm^3^ daily for 1 min for 2 weeks was prescribed, as part of the standard protocol for an MSFE procedure.

A midcrestal incision was made with vertical release incisions at the canine and tuberosity region. A full-thickness mucoperiosteal flap was elevated. The lateral maxillary sinus wall was prepared using a diamond burr with copious irrigation with sterile isotonic saline, regarding the contour of the maxillary sinus as observed on the preoperative panoramic radiograph. A bony top-hinge trap-door was mobilized and turned inward and upward into a horizontal position in the maxillary sinus, together with the carefully elevated Schneiderian membrane. The area created between the lifted lid and the sinus floor was filled only with BCP (Straumann® Bone Ceramic). The BCP was 100% crystalline, highly pure, and had a porosity of 90%. The pores were 100 to 500 μm in diameter. No membrane was used to cover the lateral window [36]. Primary wound closure was performed with Gore-Tex® sutures (W.L. Gore & Associates, Newark, DE, USA). Immediately after the procedure, a panoramic radiograph was made. Postoperative examination and removal of the sutures were performed 10 to 14 days after the MSFE procedure.

### Dental implant surgery and biopsy retrieval

**Fig. 1:**
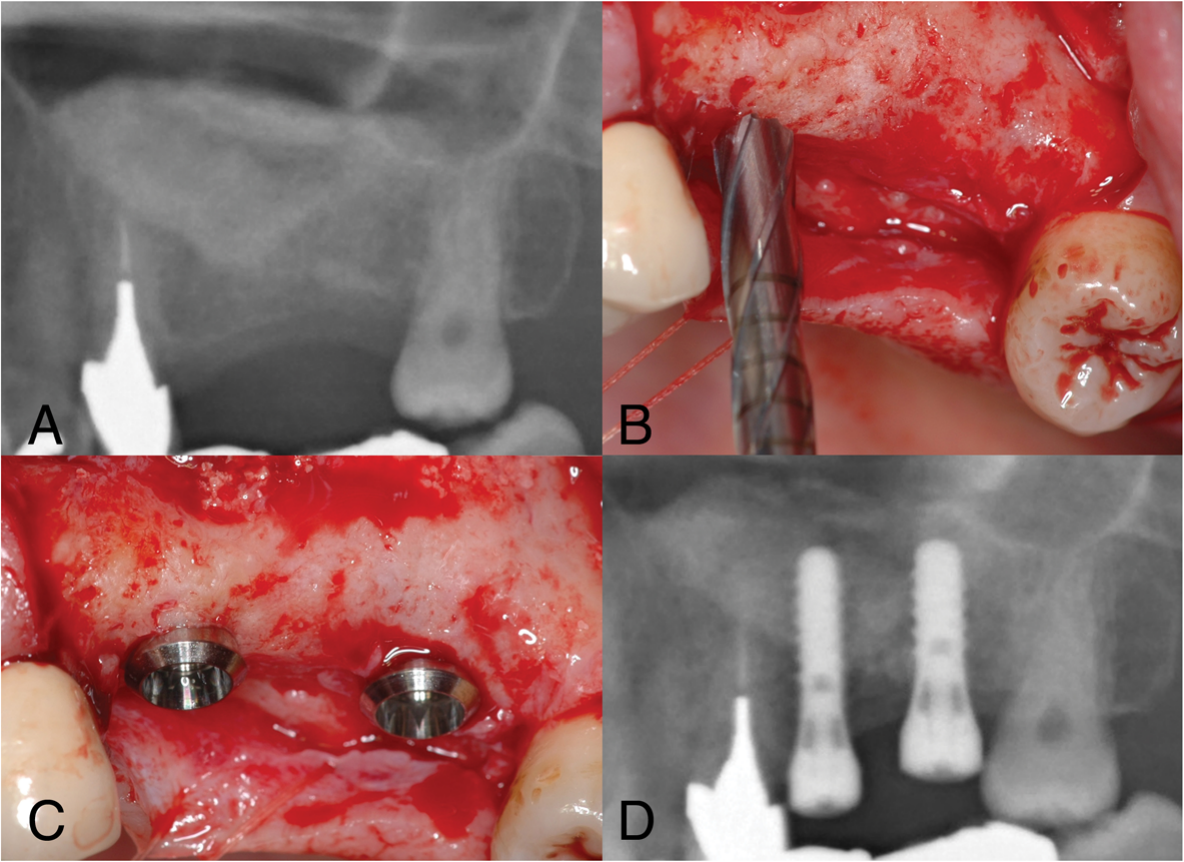
Images of patient # 5 (9-month healing time). **a.** Radiograph of the left maxillary sinus: situation 9 months after the maxillary sinus floor elevation procedure. **b.** With a trephine drill, the implant osteotomy is made and the biopsy is obtained. **c.** Clinical situation after placing two Straumann® SLA implants in the left posterior maxilla. **d.** Radiograph of two Straumann® SLA implants in the left posterior maxilla

After 9-month (five patients) and 12-month (five patients) healing times, a crestal incision was made with small mesial and distal buccal vertical release incisions. Subsequently, a full-thickness mucoperiostal flap was raised. The alveolar ridge was inspected for suitable implant placement, and the former lateral window area was inspected for tissue condition. Implant preparations were made, and biopsies were obtained from the grafted area at planned dental implant positions using trephine drills with an external diameter of 3.5 mm and internal diameter of 2.5 mm (Straumann^*®*^ trephine drill) with copious irrigation of sterile saline. In the 10 patients, 22 standard plus, regular neck, soft tissue level Straumann® SLA dental implants with a diameter of 4.1 mm and a length of 10 or 12 mm were placed (Fig. 1). The implants were left to integrate in a non-submerged unloaded fashion. Soft tissue closure was performed with Gore-Tex® sutures. A postoperative radiological examination (panoramic radiograph) was taken directly after dental implantation. Sutures were removed after 10 to 14 days and, if needed, provisional prosthetics were adapted to the new situation. Attention was paid to prevent premature loading of the dental implants. The patients were instructed to avoid loading of the posterior maxilla upon which the operation had been conducted until the 3-month integration time of the dental implants had passed and the fixed superstructures were fabricated and placed.

### Clinical evaluation

All 22 inserted dental implants were clinically tested for good primary stability. Osseointegration at abutment connection was tested with a 35-Ncm torque. One experienced oral and maxillofacial surgeon (CB) carried out all follow-up examinations.

### Radiological evaluation

Panoramic radiographs were made at patient’s intake (T0); immediately after the MSFE procedure (T1); immediately after dental implant placement (T2); 1 year after dental implant placement (T3); and 5 years after dental implant placement (T4). On the panoramic radiographs, changes in tissue height (mm) of the grafted area were measured at the implant site on the following time points: T0, T1, T2, T3, and T4. An average magnification of ×1.25 was taken into account to calculate true tissue heights.

### Qualitative histological and quantitative histomorphometric analysis

Bone biopsies were obtained during implant surgery as previously described [37]. Trephines were split and opened in order to secure the orientation of the biopsies. The biopsies were fixed overnight in 4% phosphate-buffered formaldehyde and transferred to alcohol 70% [38]. After dehydration, the bone specimens were embedded without prior decalcification in methylmethacrylate supplemented with 20% dibuthylphtalaat and 0.008 g/ml Lucidol. The biopsies were cut into 5-μm longitudinal sections (Polycut S., Leica microtome type sm2500s, Leica, Wetzlar, Germany). Bone mass indices and osteoid surface were measured in Goldner’s trichrome stained sections [39]. Tartrate-resistant acid phosphate (TRAP) staining was performed to visualize osteoclasts. Measurements were performed semi-automatically using a digitizer and image analysis software (Osteomeasure, Atlanta, GA, USA). In this study, the Von Kossa staining was used to verify remnant particles of BCP (Straumann® Bone Ceramic). BCP particles were detected semi-quantitatively by three independent observers and classified into quartiles (<25% of BCP, >25% and <50% of BCP, >50 <75% of BCP, >75% of BCP). Nomenclature was used according to the American Society for Bone and Mineral Research (ASBMR) nomenclature committee [40].

Since it was impossible to discriminate between resident and augmented bone, histomorphometric measurements were performed over the total section of the biopsy, including native and newly formed bone. The parameters were measured in consecutive fields of a complete section, in four 150-μm separated sections throughout the biopsy, covering a total measured area of 60 mm^2^. The specimens were examined for the following parameters:

Parameters evaluating vital bone mass/bone structure:

1: Vital bone volume (BV): percentage of the grafted section that is vital bone tissue (%)

2: Bone surface (BS): BS expressed as a fraction of the total vital bone volume (mm^2^/mm^3^)

3: Thickness of bone trabeculae (Tb.Th) (μm)

Parameters evaluating bone turnover:

1: Osteoid volume (OV): fraction of the vital bone tissue section that is osteoid (%)

2: Osteoid surface (OS): osteoid-covered surfaces expressed as the fraction of the total BS (%) to measure new vital bone formation

3: Osteoid thickness (O.Th) (μm)

4: Number of osteoclasts (N.Oc) per mm^2^ total area

### Statistical analysis

Because of the observational nature of this study and the limited number of biopsies, only descriptive statistics are presented. Results are expressed as mean standard deviation.

## Results

### Clinical evaluation

None of the 10 patients showed postoperative inflammation or infection after the MSFE procedure nor during surgical re-entry for dental implant placement. When opening the area for dental implant insertion, the grafted area proved to be well vascularized and the tissue at the site of the former trap-door location was slightly flexible and had a fibrous aspect. Between the periosteum and the bone graft area, adhesions were seen. Macroscopically, no voids or presence of purulent discharge were observed. Although a demarcation was observed between the grafted area and the original bone of the alveolar process, there was continuity between the grafted area and the native bone. There was no jiggling of the drill, even though bone substitute particles could still be recognized in the tissue specimen retrieved. All particles appeared well integrated in newly formed tissue. These findings were consistent in all 10 patients. In total, 22 Straumann^®^ SLA solid screw (standard plus regular neck, soft tissue level) dental implants with a diameter of 4.1 mm and a length of 10 or 12 mm were placed. Primary stability was achieved with all dental implants. All dental implants osseointegrated well and could be loaded with fixed prostheses 3 months after implant surgery. No dental implants were lost during 5-year follow-up.

### Radiological evaluation

**Fig. 2:**
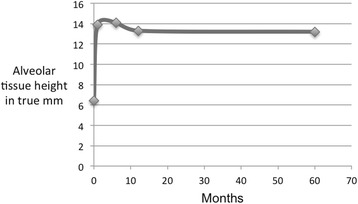
Aveolar tissue height (in true mm) over a 5-year period in the 9-month group

**Fig. 3:**
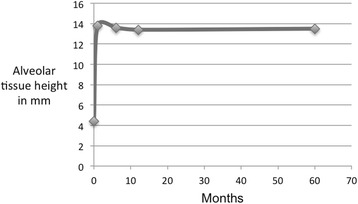
Alveolar tissue height (in true mm) over a 5-year period in the 12-month group

The increase in height of the grafted area achieved by the MSFE procedure was on an average of 7.5 mm (SD ±2.8) in the 9-month group (Table 1) and 9.3 mm (SD ±3.1) in the 12-month group (Table 2). The measured tissue height appeared to be stable between 1 and 5 years in the 9-month group (Fig. 2) and the 12-month group (Fig. 3).

**Table 1:** Alveolar tissue height measurements on panoramic radiographs (in true mm) in the 9-month group

Patient	Gender/age	Implant site	T0	T1	Increase	T2	T3	T4
1	F/54	15	5.4	15.2	9.8	14.0	13.6	13.2
1	F/54	16	5.7	14.3	8.6	14.6	13.0	13.5
2	M/62	16	5.5	15.1	9.6	13.2	12.4	12.4
3	F/64	15	7.0	15.2	8.2	13.4	12.8	12.2
4	M/63	26	6.0	11.7	5.7	14.7	14.8	14.6
4	M/63	27	10.0	11.3	1.3	14.8	13.5	13.4
5	M/40	26	4.1	12.3	8.2	12.4	12.0	12.5
5	M/40	27	7.3	16.3	9.0	15.5	14.3	13.8
Mean	56.6		6.4	13.9	7.5	14.1	13.3	13.2
SD			1.7	1.9	2.8	1.0	0.9	0.8

Age in years at biopsy retrieval; hard tissue height corrected for magnification (×1.25) on panoramic radiograph

*M* male, *F* female, *T0* (native bone height) preoperative alveolar bone height, *T1* directly after MSFE procedure, *T2* immediately after dental implant placement, *T3* 1 year after dental implant placement, *T4* 5 years after dental implant placement

**Table 2:** Radiological results (alveolar tissue height measurements in true mm) in the 12-month group

Patient	Gender/age	Implant site	T0	T1	Increase	T2	T3	T4
1	F/53	15	6.3	17.6	11.3	18.7	17.4	17.4
1	F/53	16	2.5	17.6	15.1	17.9	16.4	15.9
1	F/53	17	1.3	15.5	14.2	13.7	12.8	12.6
2	F/53	26	8.8	17.2	8.4	16.7	16.1	17.0
2	F/53	27	7.9	13.1	5.2	11.3	12.0	11.9
3	F/67	14	4.2	13.4	9.2	14.1	14.0	14.0
3	F/67	15	4.1	11.0	6.9	9.9	12.7	12.8
3	F/67	16	5.6	9.9	4.3	9.0	11.4	11.0
4	M/67	14	2.5	13.9	11.4	13.8	13.3	14.1
4	M/67	15	3.0	12.2	9.2	12.5	11.2	11.8
4	M/67	17	4.3	10.7	6.4	10.5	10.1	10.3
5	F/51	24	5.5	13.7	8.2	14.0	13.8	14.4
5	F/51	25	2.5	13.0	10.5	14.6	13.6	13.2
5	F/51	26	3.6	14.1	10.5	14.0	13.2	13.4
Mean	58.2		4.4	13.8	9.3	13.6	13.4	13.5
SD			2.0	2.3	3.1	3.8	2.0	2.1

Age in years at biopsy retrieval; hard tissue height corrected for magnification (×1.25) on panoramic radiograph

*M* male, *F* female, *T0* (native bone height) preoperative alveolar bone height, *T1* after MSFE procedure, *T2* immediately after dental implant placement, *T3* 1 year after dental implant placement, *T4* 5 years after dental implant placement

### Qualitative histological evaluation

The histological evaluation was performed on the complete section, comprising native bone, newly formed bone, and residual graft material. The BCP particles were surrounded by connective tissue, osteoid islands, and newly formed bone. From the residual bone in all specimens, new bone formation was detected following the scaffold of the bone substitute, starting in cranial direction. This newly formed bone consisted of woven bone as well as lamellar bone and appeared as vital bone tissue containing osteoblasts, osteoid covering the border, and osteocytes inside bone lacunae. Cranially, near the lifted trap-door, some osteoid islands with osteogenic activity were detected.

**Fig. 4:**
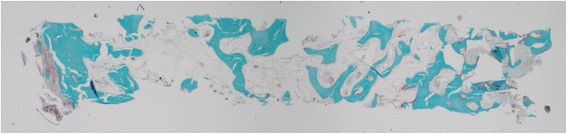
Patient # 1 (12-month healing time): overview of a typical example of a bone biopsy stained with Goldner trichrome staining (magnification ×10)

**Fig. 5:**
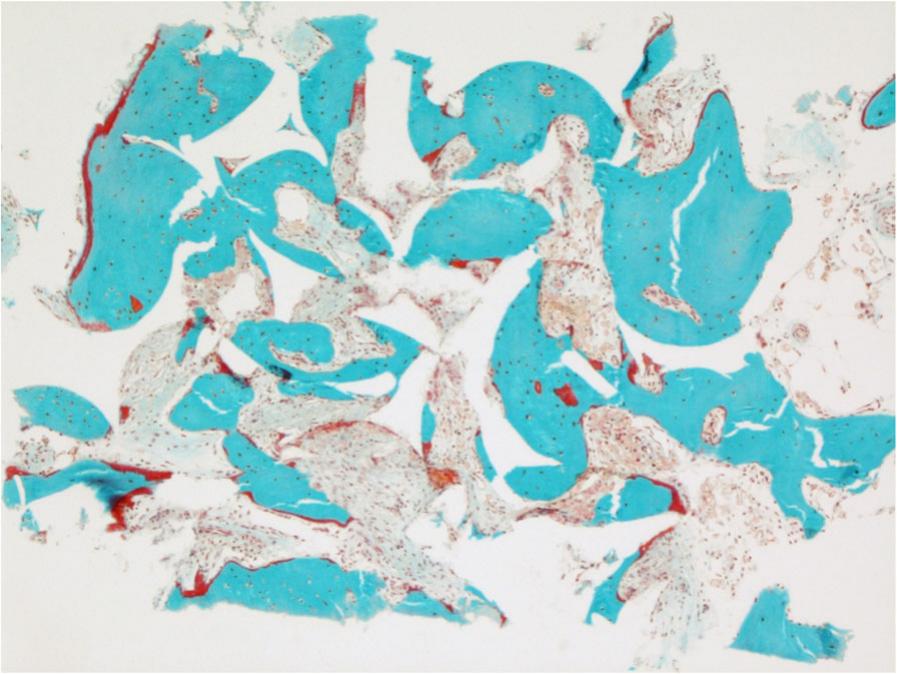
Patient # 4 (9-month healing time): increased bone formation following the shape of the grafted particles stained with Goldner trichrome staining (magnification ×100)

**Fig. 6:**
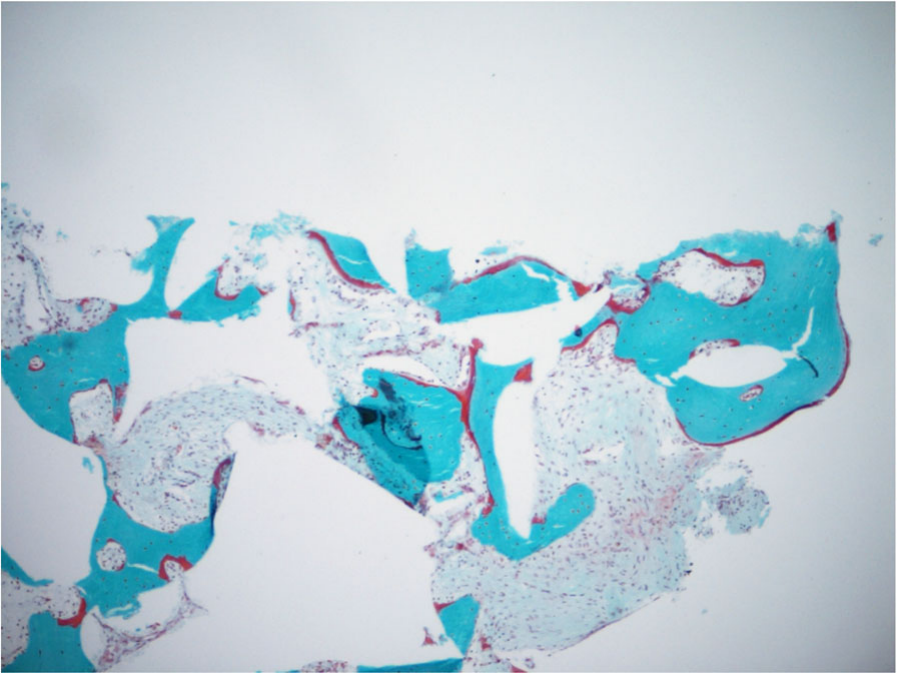
Patient # 1 (12-month healing time): increased bone formation following the shape of the grafted particles that are still present (magnification ×100)

Histological observations did not show inflammatory cells in the tissue adjacent to the bone substitute particles. Bone marrow-like tissue, which included blood vessels, was observed in between the bone trabeculae (Fig. 4). Reinforcement by lamellar bone was shown in some areas after 9 and 12 months (Figs. 5 and 6). No Howship’s lacunae could be detected on the characteristic outlines of the substitute particles. Fragments of the substitute particles were present in the sections of the 9-month group and the 12-month group, as confirmed by Von Kossa staining. Regardless of the histological process, the contours of the bone substitute remnants were clearly detectable which enabled analyses.

### Quantitative histomorphometric evaluation

In the 9-month group, the average vital bone volume was 35.2% of the total biopsy volume (SD ±9.5) of which 8.8% (SD ±3.8) was osteoid. The osteoid surface covered 42.4% (SD ±12.1) of the bone surface. The BS covered 4.2 mm^2^/mm^3^ (SD ±1.9). In the total area for the 12-month group, the average vital bone volume was 28.2% of the total biopsy volume (SD ±3.2) of which 3.4% (SD ±2.5) was osteoid. The osteoid surface covered 8.2% (SD ±5.3) of the bone surface. The BS covered 8.3 mm^2^/mm^3^ (SD ±1.3). In conclusion, vital bone volume and bone turnover decreased in the 12-month group compared to the 9-month group. An overview of the individual histomorphometric findings is listed in Tables 3 and 4.

**Table 3:** Histomorphometric evaluation of the biopsies after a 9-month healing time

Patient (*N*)	Gender/age)	Retrieval location	BV/TV (%)	BS/TV (mm^2^/mm^3^)	Tb.Th (μm)	OV/BV (%)	OS/BS (%)	O.Th (μm)	N.Oc/Tar (1/mm^2^)
1	F/54	15	51.3	2.3	461.3	5.4	43.4	28.3	0.8
2	M/62	16	30.4	2.2	278.6	13.3	53.3	331.8	2.0
3	F/64	15	29.3	4.6	121.7	8.5	50.0	10.2	2.5
4	M/63	27	36.3	5.8	168.8	4.7	22.3	18.8	2.3
5	M/40	26	28.5	6.2	93.0	12.0	43.0	80.2	5.0
Mean			35.2	4.2	224.7	8.8	42.4	93.9	2.5
SD			9.5	1.9	150.0	3.8	12.1	135.8	1.5

Not intact biopsies were excluded from histomorphometric examination. Age in years at biopsy retrieval

*M* male, *F* female, *BV/TV* vital bone volume/total volume, *BS/TV* bone surface/total volume, *Tb.Th* trabeculae thickness, *OV/BV* osteoid volume/vital bone volume, *OS/BS* osteoid surface/bone surface, *O.Th* osteoid thickness, *N.Oc/Tar* number of osteoclasts in total area

**Table 4:** Histomorphometric evaluation of the biopsies after a 12-month healing time

Patient (*N*)	Gender/age	Retrieval location	BV/TV (%)	BS/TV (mm^2^/mm^3^)	Tb.Th (μm)	OV/BV (%)	OS/BS (%)	O.Th (μm)
1	F/53	16	25.5	6.9	74.1	1.7	5.2	12.8
2	F/53	25	30.8	9.2	66.8	1.1	3.1	12.7
2	F/53	26	29.3	9.5	61.5	4.2	9.2	14.7
3	M/67	17	24.0	7.5	64.4	6.6	15.2	14.0
Mean			28.2	8.3	66.7	3.4	8.2	13.6
SD			3.2	1.3	5.4	2.5	5.3	1

Not intact biopsies were excluded from histomorphometric examination. Age in years at biopsy retrieval

*M* male, *F* female, *BV/TV* vital bone volume/total volume, *BS/TV* bone surface/total volume, *Tb.Th* trabeculae thickness, *OV/BV* osteoid volume/vital bone volume, *OS/BS* osteoid surface/bone surface, *O.Th* osteoid thickness, *NOc/BPm* not measured as an insignificant number of osteoclasts were available

## Discussion

This study presents the clinical, radiological, histological and histomorphometric results on the use of a biphasic calcium phosphate (Straumann**®** bone ceramic) in a MSFE procedure with healing times of 9 and 12 months. During the clinical evaluation, it appeared that both 9-month and 12-month healing times resulted in integration of the grafted BCP with the original maxillary bone (sinus floor), which was stable enough to ensure successful dental implant placement. It should be mentioned that in this study, a minimal native alveolar bone height of 4 mm was preferred, ensuring a certain primary stability of the dental implants placed. An adequate and stable tissue height in the grafted area was observed radiologically in a 5-year follow-up in all patients in both 9-month and 12-month healing time groups.

Radiological observations show very stable results in different healing times, in a previous 6-month study [27] and after 9- and 12-month healing times in the present study. However, this does not reveal the actual vital bone height available for attachment to the dental implant surface. This can only be measured by histological investigations. Reviews show that the loss of dental implants with an intra-osseous length of 8 mm or more, placed in native bone, is minimal [4]. Previously, the histomorphometrical and histological evaluation, 6 months after an MSFE procedure, using Straumann**®** bone ceramic was reported with a 1-year follow-up. At that time, no loss of dental implants was reported. In the present study, none of the implants in the 9- and 12-month groups were lost. Histological investigation showed that mineralized bone tissue was observed to be in intimate contact with the bone substitute particles, indicating that the graft material possesses osteoconductive properties [27] (which is in agreement with other observations) [16, 24, 34]. This positive effect might be explained by its chemical composition. BCP materials have shown bone formation simultaneously with material degradation [24, 25]. BCP exhibited moderate signs of substitute degradation in humans not only after 6 months, as previously reported by Frenken et al. [27]. The present study still observed remnants of BCP after 9 and 12 months which suggests that the ossification rate is not the same as the resorption rate of the BCP. Because osteoclasts were detected next to the characteristic outlines of the substitute particles, it is suggested that BCP is resorbed by osteoclasts. The high bone formation in the newly formed bone area indicates that after 12 months, bone cells are still actively forming new bone matrix, thereby absorbing and replacing BCP in vital bone tissue.

In the cranial part of the biopsy, some osteoid islands with osteogenic activity were detected, possibly caused by osteoinductive properties from the lifted bony trap-door. In the present study, histomorphometric analyses revealed that the vital bone volume was higher in the 9-month healing time group than in the 12-month healing time group, while one would expect to find more newly formed bone in time as more of the bone substitute resorbs. As the mean original native alveolar bone height is 6.4 mm in the 9-month group and 4.4 mm in the 12-month group and augmented portion of the 12-month group (9.3 mm) is higher than the 9-month group (7.5 mm), this may have a negative impact on the relatively smaller portion of the bone volume of the total biopsy in the 12-month group. This is a limitation of the present study.

However, the average 2 mm of difference in the native bone height between the 9- and 12-month groups does not fully explain the difference in BT/TV that was found between the two groups. Furthermore, the thickness of the bone trabeculae decreased suggesting that at 12 months, bone turnover returns to a relatively normal bone remodeling status, indicative of a new balance in bone tissue. However, woven bone (data not shown) and the remnants of the BCP were still present at 12 months, contradicting this hypothesis. Nevertheless, from a histological and histomorphometric perspective in the present study, a 9-month healing time may be the optimal time for the placement of dental implants. Although the sample size of the two groups is small, multiple dental implant placements deliver sufficient data for evaluation. The 12-month period from MSFE to implant placement(s) is considered to be a long time. Most patients are not willing to wait that long, which makes these bone samples very scarce and therefore valuable for long-term observations. The implication of the small sample size is that this study has an observational nature and, therefore, only descriptive statistics are presented.

## Conclusions

Based on clinical, radiological, histological, and histomorphometric analysis, this study confirms the suitability of BCP for vertical augmentation of the posterior maxilla by means of an MSFE procedure, allowing dental implant placement after 9 and 12 months healing times. Yet, complete degradation of the BCP particles does not occur within a 12-month healing time. From a histological and histomorphometric perspective, a 9-month healing time for this type of BCP may be the optimal time for the placement of dental implants.
